# Outcomes of “Over the Top” Anterior Cruciate Ligament Reconstruction Associated with a Lateral Extra-Articular Tenodesis in Children

**DOI:** 10.3390/jcm13051501

**Published:** 2024-03-05

**Authors:** Abel Gomez-Caceres, Iskandar Tamimi-Mariño, Francisco Javier Martinez-Malo, Raphael Pierre Idiart-Charrier, Ignacio Vieitez-Riestra, Ivan Medina-Porqueres

**Affiliations:** 1 HM Hospital, 29010 Malaga, Spain; 2 Department of Physical Therapy, Faculty of Health Sciences, University of Malaga, 29071 Malaga, Spain; 3 Regional University Hospital, 29010 Malaga, Spainjaviermmalo@gmail.com (F.J.M.-M.);; 4 Vithas Xanit International Hospital, 29630 Malaga, Spain; raphael.idiart@gmail.com

**Keywords:** growth disturbances, ACL reconstruction, adolescents, knee ligaments, autografts, Lemaire procedure, skeletal immaturity

## Abstract

(1) **Purpose**: The incidence of anterior cruciate ligament (ACL) ruptures in children and adolescents has considerably increased during the last decades due to higher levels of competitive athletic activity, and early sport specialization and professionalization. Contemporary ACL reconstruction techniques have recently been subject to renewed interest in this population. The objective of this study is to report the short- and mid-term results of our physis-sparing ACL reconstruction technique using an “over the top” technique associated with a modified Lemaire procedure. (2) **Methods**: A retrospective series of 12 junior soccer players who presented to our clinic with a torn ACL between January 2019 and September 2021 was reviewed. The inclusion criteria were patients under 15 years with open tibial and femoral physes, with a stable contralateral knee, a minimum follow-up of 6 months, and a time frame from injury to surgery of <3 months. Patients with previous knee surgery, structural concomitant injuries, muscular, neurological, or vascular abnormalities, or hypersensitivity to metal alloys were excluded. The functional evaluation was performed using the International Knee Documentation Committee (IKDC) rating, Lysholm score, and Tegner activity level. Moreover, clinical and radiological assessments were also performed, including KT-1000 and knee X-rays. (3) **Results**: We identified 1 female and 11 male patients with ACL tears, with a mean age of 13.17 ± 0.9 months. Concomitant injuries include isolated vertical and bucket-handle tears of the medial meniscus, lateral meniscus tears, bilateral tear of both menisci. The mean follow-up time was 26 ± 12.6 months. The average IKDC, Lysholm and Tegner scores were 93.29 ± 11.04, 95.08 ± 13.2 and 9 ± 0.0 points, respectively. The average KT-1000 score of the participants was 0.96 ± 1.6 points. None of the included patients reported post-surgical complications or required additional surgeries. (4) **Conclusions**: Our novel ACL reconstruction with LET technique is a safe procedure that resulted in good clinical outcomes, lower failure rate and return to sports in skeletally immature patients.

## 1. Introduction

The number of knee injuries in growing children has increased considerably in recent years due to higher levels of competitive athletic activity, and early sport specialization and professionalization [1]. Anterior cruciate ligament (ACL) tears are one the most common injuries in this age group, and they are often associated with other injuries such as meniscal and chondral injuries [2].

The incidence of ACL injuries in children under 15 years of age is 15 per 100,000 in exposed athletes. This rate has been reported to be increased by 924% between 1994 and 2006, based on the National Survey of Ambulatory Surgery and National Hospital Discharge Survey databases, with adolescents as the largest per capita cohort of patients undergoing ACL repairs [3]. The number of ACL reconstructions performed on patients aged 18 years or younger in pediatric hospitals in USA markedly increased by nearly 3 times relative to orthopedic surgeries between 2004 and 2014 [4].

A clinical decision should be made on whether a conservative approach should be advocated until skeletal maturity is reached or surgery can provide an adequate functional outcome without damaging the physis. In the past, nonoperative management relying on neuromuscular conditioning, activity modification, and bracing until skeletal maturity followed by delayed surgical repair were once the standard of care for ACL tears in growing patients [5]. However, surgical treatment has prevailed over conservative treatment in recent years since there is a higher risk of chondral and meniscal injuries and future disability in patients with ACL deficiency who are treated conservatively. Consequently, current practice has evolved to favor early surgery in order to reduce the number of associated injuries and improve the rate of return to sports practice for this challenging patient population [6,7,8].

Regarding the surgical treatment of these injuries, we must bear in mind that these patients are in a growing age. Therefore, the physis should be spared to avoid potential growth deformities or disturbances such as tibial recurvatum due to tibial tubercle apophyseal arrest, the formation of physeal bone bridges along with accelerated growth with leg length discrepancy (LLD) and/or angular deformity due to physeal arrest, retardation, tethering, or overgrowth [9]. Accordingly, many “physeal-sparing” and “physeal-respecting” surgical techniques and treatment algorithms have been described to manage ACL injuries, based on age, gender and the state of the growth plate. Physeal-sparing, partial transphyseal, and transphyseal-sparing techniques are among the most popular procedures, with similar clinical outcomes; however, physeal-sparing techniques provide better restoration of knee laxity [10].

“Over the top” techniques with autologous hamstring tendon grafting have been used for the treatment of ACL injuries. These reliable, single-stage procedures combine intra- and extra-articular techniques, eliminating the need for extensive femoral tunnel osteolysis, and ensuring an isometric placement of the graft, with lower risk of tunnel convergence. “Over the top” techniques have few complications involving the growth and angulation of the operated knee, restore joint stability and the biomechanics of the knee and have a similar re-operation rate and clinical outcomes compared to epiphyseal [11,12,13,14].

On the other hand, due to their excellent results, the use of lateral reinforcements, such as associating a lateral extra-articular tenodesis (LET) to an ACL reconstruction, is progressively becoming more popular, even in patients with skeletally immature and “over the top” techniques [14,15]. Such extra-articular procedures allow for the enhancement of the stability achieved with the reconstruction itself, reducing the risk of recidivism in high-risk subjects. Data from biomechanical studies give the anterolateral complex (ALC) a role as a secondary stabilizer to the ACL in opposing anterior tibial translation and internal tibial rotation, which may offload forces experienced by an ACL graft during pivoting activities.

However, information pertaining to the application of LET in conjunction with “over the top” techniques in a population of children patients with open physis is scarce. This paper attempts to find a simpler and more effective method to treat ACL total rupture, which enhances the stability achieved with the reconstruction itself, and, at the same time, spare the subject’s physis and provide a compensating anterolateral rotatory stability, reducing the risk of recidivism. Therefore, the objective of this study was to report the short- and mid-term results of our physis-sparing ACL reconstruction technique using an “over the top” technique associated with a modified Lemaire procedure in skeletally immature children and adolescents.

## 2. Materials and Methods

### 2.1. Study Population

For the purposes of this study, a retrospective series of 12 junior soccer players who presented to our clinic with a torn ACL between January 2019 and September 2021 was reviewed. The inclusion criteria were as follows: (i) patients under 15 years with open tibial and femoral physis at the time of the surgery; (ii) asymptomatic, stable, and functional contralateral knee; (iii) a time frame from injury to surgery of <3 months; and (iv) minimum follow-up of 6 months. Those patients with a history of knee surgery of the contralateral and/or ipsilateral knee, hypersensitivity to cobalt, chromium, or nickel, and muscular, neurological, or vascular abnormalities were excluded. Further exclusion criteria included concomitant multiligament injuries, full-thickness cartilage lesions and fractures.

This study was conducted following the ethical principles for medical research involving human subjects of the World Medical Association [16] and it was approved by the local Ethical Board Committee from the Institute of Biomedical Research of Malaga (protocol code: 2022-002624-22, date 26 May 2022). All the participants/their legally authorized representative signed a consent form to participate in this study. Demographic data, surgical details, concomitant diagnoses, and outcome measures were collected for each patient.

### 2.2. Surgical Technique

**Figure 1:**
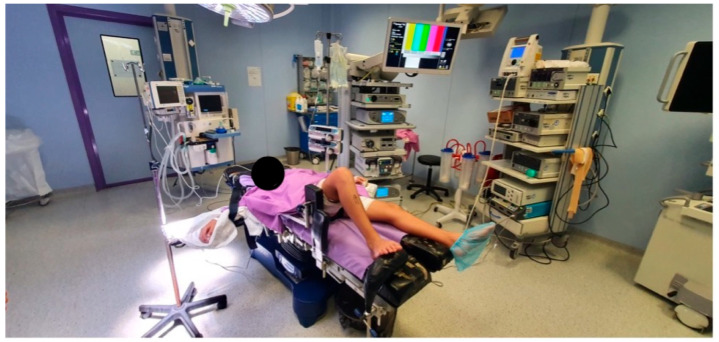
Position of the patient in the operating room. The patient is placed in the supine position. A tourniquet is placed on the proximal thigh, and an L-shaped support is placed on the distal end of the table to keep the knee in about 80º of flexion. Two additional supports are placed laterally.

All the patients were subjected to general anesthesia and placed in the supine position. A tourniquet was placed on the proximal thigh, and an L-shaped support was placed on the distal end of the table to keep the knee in about 100° of flexion. Two additional supports were placed laterally (Figure 1). Standard anterolateral viewing and anteromedial working arthroscopy portals were made to perform a full exploration of the knee. Then, any meniscal injuries were identified and repaired using Truespan TM (Depuy-Synthes, Raynham, MA, USA) implants. The ACL tear was confirmed under direct visualization and a debridement of any remnant tissues was performed using a No. 4 arthroscopy shaver (Depuy-Synthes, Raynham, MA, USA). Then, the exit point of the tibial tunnel was marked with an electrocautery device (Depuy-Synthes, Raynham, MA, USA).

**Figure 2:**
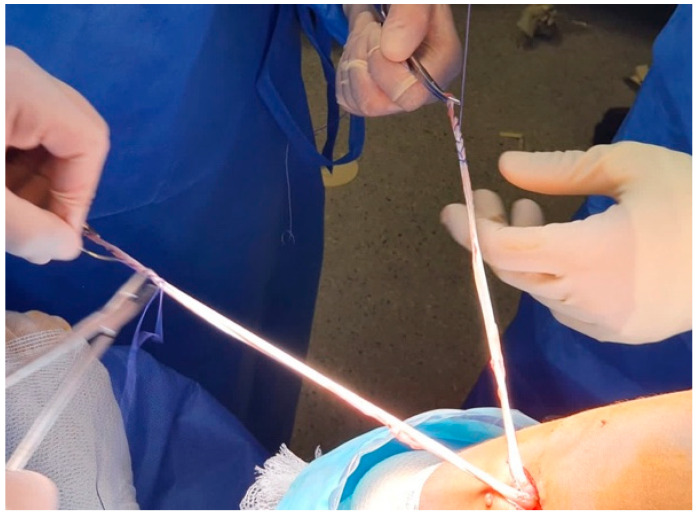
Hamstring tendon graft. The hamstring tendons are sutured independently without releasing their distal disinsertions (right knee).

Through an oblique incision on the medial aspect of the tibia, above and in the same direction of the hamstrings tendons, the gracilis and semitendinous tendons were then extracted using a tenotome without releasing their distal disinsertions. Next, the tendons were cleaned from any remaining muscle fibers, and the ends of both tendons were sutured with a number 2 high-resistance thread (Figure 2). Finally, we measured the diameter of the double-bundle autograft to create the tibial tunnel.

**Figure 3:**
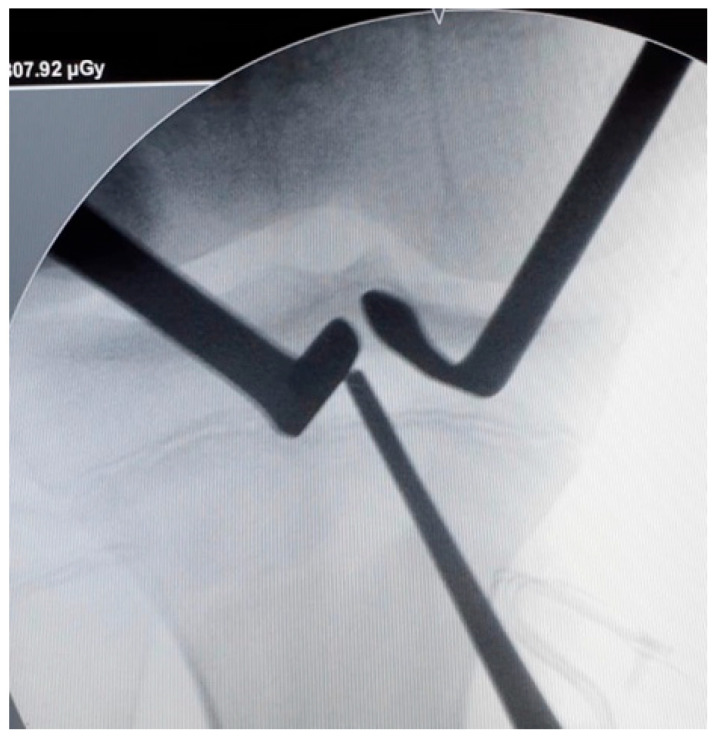
Tibial tunnel positioning. The tibial tunnel is performed under radiological control. A guide-pin is inserted proximal to the tibial physis (right knee).

The tibial guide was then centered over the ACL tibial footprint under arthroscopic vision and an image intensifier (Figure 3). A guide pin was inserted proximal to the tibial physis and drilled in an outside-in direction. The angulation of the tibial guide varied depending on the position of the physis. The size of the tunnel was determined by the diameter of the double-bundle graft.

A slight opening was then made through the posterolateral capsule of the knee and Kocher-type forceps were employed to pass a suture “over the top” to the anteromedial portal from that posterolateral opening. To conclude, the suture itself was used to pass the graft to the posterolateral window after retrieving it through the tibial tunnel.

### 2.3. Modified Lemaire

**Figure 4:**
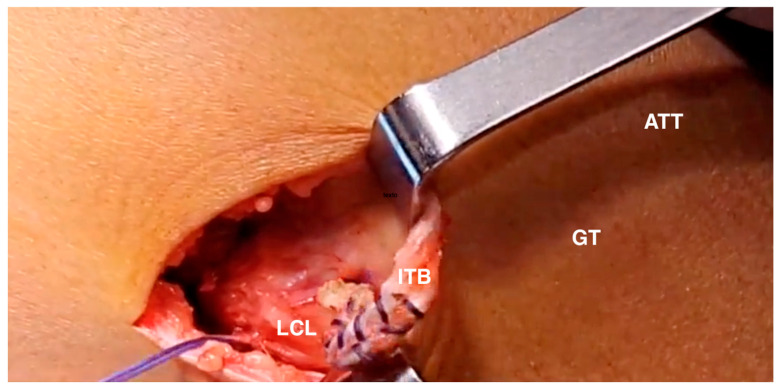
Modified Lemaire technique. The plasty of the iliotibial tract is passed under the lateral collateral ligament (right knee). LCL: lateral collateral ligament; LLE: lateral epicondyle; GT: Gerdy's tubercle; ATT: anterior tibial tuberosity; ITB: iliotibial band.

A 3–4 cm lateral incision was made over the lateral epicondyle and a 1 cm-wide band was separated from the iliotibial tract, 4–5 cm proximal to the lateral epicondyle. The band was then extended distally to Gerdy´s tubercle in order to obtain the maximum length possible. A high-strength FiberWire suture was then employed to pull the free end and pass it under the lateral collateral ligament (Figure 4).

**Figure 5:**
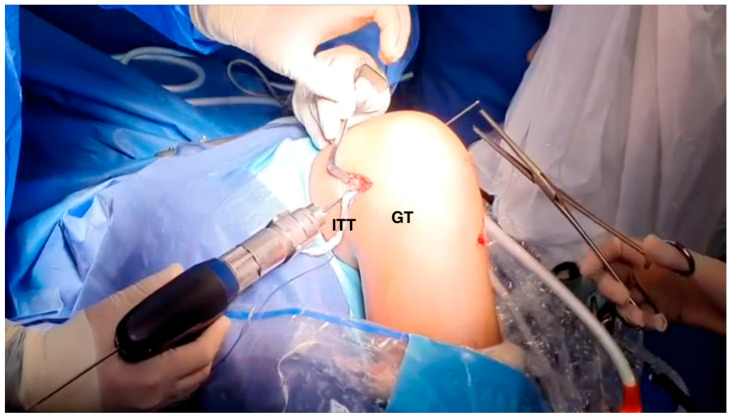
Femoral tunnel drilling (right knee). A pin guide is inserted proximal and posterior to the distal femoral physis from lateral to medial. ITT: iliotibial tract. GT: Gerdy’s tubercle.

**Figure 6:**
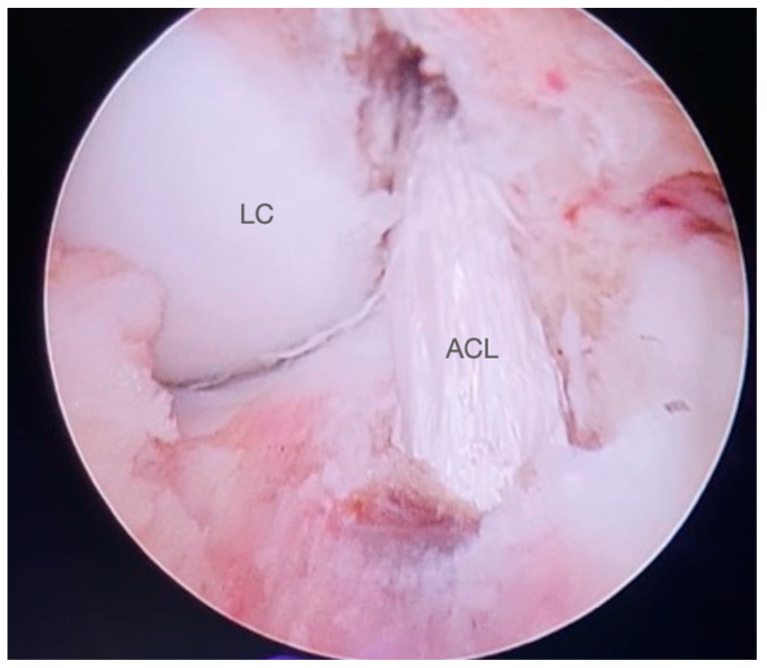
Final appearance of the intra-articular ACL graft (right knee). LC: lateral condyle. ACL: anterior cruciate ligament.

Finally, under image intensifier guidance, a proximal femoral tunnel was drilled posterior to the distal femoral physis, from lateral to medial (Figure 5). The dimensions of the femoral tunnel were dependent on the combined diameter of the iliotibial Lemaire´s graft and the double-bundle hamstring graft. Both grafts were then fixed within the tunnel with an interference screw employing full extension and neutral rotation components (Figure 6). A detailed description of the surgical procedure has been previously described elsewhere [17].

### 2.4. Postoperative Protocol

Patients were instructed to undertake mobilization exercises of the toes, ankle, knee, and hip every waking hour and were encouraged to start flexion-extension exercises in the early postoperative period. If a meniscal repair was performed, patients were advised to avoid knee flexion beyond 90°. Isometric exercises of the dynamic knee-stabilizing muscles were initiated from the first postoperative day. Patients were kept partially weight-bearing with crutches for 6 weeks. Gradual progression to more functional activities was planned according to each patient’s progress, and patients gradually returned to their normal activities thereafter, depending on the strength and neuromuscular coordination levels. Return to competition was permitted after at least 15 sessions of full and unrestricted training, but never before 9 months after surgery.

### 2.5. Follow-Up

All patients were evaluated after surgery every week up to the first postoperative month, monthly up to 3 months, and annually thereafter. At least 12 months after the index procedure, the patients were contacted for evaluation to assess the clinical status. The functional evaluation was performed using the International Knee Documentation Committee (IKDC) rating, Lysholm score, and Tegner activity level. Moreover, clinical and radiological assessments were also performed, including the knee arthrometer test (KT-1000 knee arthrometer, Medmetric Corp, San Diego, CA, USA) and knee X-rays. The IKDC and Lysholm were scored 0 to 100, with 0 expressing the lowest level of function or highest level of symptoms, and 100 points indicating the highest level of function and lowest level of symptoms. The Tegner activity level graded activity based on work and sports activities on a scale of 0 to 10, where zero represents disability because of knee problems and 10 represents national- or international-level soccer. Patient demographic data, concomitant surgical procedures and perioperative complications were recorded. Demographics included gender, age, joint affected (Table 1). Return to sport-specific activity was allowed once muscle strength and neuromuscular control were reestablished, but never before 9 months.

The functional evaluation was performed using the International Knee Documentation Committee (IKDC) rating, Lysholm score, and Tegner activity level. Moreover, clinical and radiological assessments were also performed, including the knee arthrometer test (KT-1000 knee arthrometer, Medmetric Corp) and knee X-rays.

### 2.6. Statistical Analysis

Due to the fact that the study was not powered to allow for inferential statistical comparisons within groups, the focus of the analysis was on identifying possible trends across them by standard descriptive statistics. These descriptive statistics were calculated to characterize the patient population, with continuous variables reported as means and standard deviations, and discrete variables reported as frequencies and percentages. The difference in ACL laxity between operative knee and contralateral knee was defined as measured laxity in the operative knee minus measured laxity in the contralateral knee through KT-1000 evaluations. The pre-to-post difference in anterior tibial displacement was compared using a paired sample *t*-test. Correlation was performed to assess whether results from KT-1000 evaluations corresponded to reported functional status through IKDC and Lysholm scores at the final follow-up. The statistical analysis was performed using the statistical analysis system IBM SPSS Statistics 20 (IBM Inc., Chicago, IL, USA). A *p* value of 0.05 was considered significant.

## 3. Results

We identified 1 female and 11 male patients with ACL tears, with a mean age of 13.17 ± 0.9 months. In addition, two patients sustained bucket-handle tears of the medial meniscus, two other patients had lateral meniscus tears, one patient suffered a tear of the medial and lateral menisci, and a sixth patient had an isolated vertical medial meniscus tear. At the time of the surgery all patients were amateur soccer players. The mean follow-up time was 26 ± 12.6 months. Baseline patient characteristics are gathered in Table 1.

The average IKDC, Lysholm and Tegner scores were 93.29 ± 11.04, 95.08 ± 13.2 and 9 ± 0.0 points, respectively. The average KT-1000 of the participants was 0.96 ± 1.6 points. At the time of the follow-up evaluation, the postoperative KT-1000 arthrometer side-to-side difference averaged 0.72 ± 1.51 mm. There were no significant correlations between KT-1000 evaluations and IKDC (r = −0.329, *p* = 0.296) and Lysholm scores (r = −0.327, *p* = 0.299). At clinical assessment, one patient was found to have a + 1 positive Lachman with a firm endpoint, a second patient had a slight pivot shift and a + 1 anterior drawer test and a third patient had a + 1 anterior drawer. None of the included patients reported post-surgical complications or required additional surgeries (Table 2).

## 4. Discussion

This study evaluated clinical and patient-reported results of skeletally immature patients who underwent a particular physis-sparing ACL reconstruction method using an “over the top” technique associated with a modified Lemaire procedure. At the 14-month follow-up, our series demonstrated favorable clinical and functional outcomes, no surgical complications or growth plate disturbances, and no retears or additional surgeries. Taken together, our findings suggest that concomitant physeal sparing ACL reconstruction and the modified Lemaire´s procedure is safe and efficient, with favorable clinical and functional outcomes among skeletally immature patients and little risk of plasty rupture.

Young patients who perform high-impact sports are at a higher risk of ACL reconstruction failure [18]. Graft failure has been estimated to occur in 17% of cases in skeletally immature populations at an average time of 13.6 months, and can be confirmed by clinical examination or imaging studies [19]. Potential factors associated with failure were graft choice, with soft tissue grafts twice as likely to fail compared with patellar tendon grafts (13% vs. 6%) as well as interactions contributed by the ACL reconstruction technique and growth plate immaturity. Therefore, surgeons should use all the available means to reduce the odds of graft failure in these patients. Accordingly, the anterolateral reinforcement has been shown to be the most relevant additional surgical gesture that may reduce the re-rupture rates in these patients. The reinforcement of an ACL reconstruction with an anterolateral ligament (ALL) reconstruction in adults has been observed to better restore the kinematics and rotational stability of the knee [20]. Moreover, this improvement may also be extrapolated to child cohorts, reducing their cumulative failure rate and improving knee objective stability with no increase in perioperative complications [21].

Managing pediatric ACL injuries requires understanding the mechanisms of bone growth and physeal injury, as well as the natural history of the delayed versus acute surgical approach. Surgeons must be familiar with the different surgical alternatives that are available, and the risks and benefits associated with their performance. Previous research has shown that the use of extraphyseal techniques has not been related to higher rates of growth abnormalities, reconstruction failures, or worse clinical outcomes [10]. However, these procedures may result in poorer stability and kinematic restoration of the knee [22].

In this study, all our patients were able to return to their previous sport after surgery. These results are higher than those published by Kay et al. in their meta-analysis, where the pooled data from 20 studies suggested a 92% rate of return to sport following ACL reconstruction in children and adolescent athletes [20]. Only 2 of the 12 patients in our series did not achieve excellent scores in the Lysholm, Tegner and KOOS tests. However, one of these two patients suffered from Osgood-Schlatter disease, which could have hindered his clinical outcome. The second patient had a Lysholm score of 86, and negative Lachman, anterior drawer, and Pivot Shift tests. All the patients had a full range of movement of the operated knee. Moreover, differences in laxity compared to the contralateral leg measured with a KT-1000 device were comparable to previous research, and the presented technique achieved high grades of knee stability. Accordingly, at the time of the clinical assessment, half of the patients had knee stability equal or superior to the contralateral joint, and only one patient had a slightly positive Pivot Shift test. These results could be explained by the extra-articular lateral tenodesis, which may have improved the rotational stability of the knee. Moreover, early ACL reconstruction in those young and active patients might contribute to the prevention of secondary meniscal or chondral lesions.

Nevertheless, a significant limitation of our technique could be the diameter of the graft. In our study, the diameter of the graft ranged between 6 and 7 mm. A graft size lower than 7–8 mm has been associated with higher re-ruptures rates [23]. In addition, graft diameters of less than 8 mm have been related to higher revision rates in populations aged 20 years or younger [24,25]. According to Spragg et al., the likelihood of a revision is 0.82 lower for each increase of 0.5 mm in diameter within the 7.0 to 9.0 mm range [26]. In contrast, in their recent study, Helito et al. found no difference in the rate of retear, anteroposterior translation with KT-1000, degree of pivot-shift, and scores such as the Lysholm and Subjective IKDC, comparing a group of patients with goose grafts greater than 7 mm and another group with grafts less than or equal to 7 mm associated with reconstruction of the ALL [27].

An important aspect of the recovery process of patients undergoing ACL reconstructions is the integration and ligamentization of the graft. Not disinserting the hamstring tendons maintains the graft´s vascularization and innervation and improves the ACL graft proprioception, as elucidated by Zaffagnini et al. in their cadaveric study [28]. In a study performed on rabbits, Papachristou et al. concluded that maintaining graft insertion maintains its viability. This avoids the initial phase of graft necrosis, which leads to a more rapid ligamentization [29]. Grassi et al. and Zaffagnini et al. reported “over the top” techniques using a hamstring autograft without disinserting its distal insertions and passing the graft through a tibial tunnel. However, these authors fixed the ACL graft with metallic staples and used the remaining graft to reconstruct the ALL. This technique has achieved good clinical outcomes at 10–20 years of follow-up without increasing the risk of causing osteoarthritis of the lateral compartment of the knee [13,30]. Different MRI-based studies have shown that patients in whom the distal insertion of the graft was preserved, a better remodeling was achieved. Moreover, other reports have shown lower reoperation rates, better functional outcomes and shorter recovery periods [31,32]. In light of these data, the hamstring tendons were not disinserted in any patient in our series.

Current research suggests that young athletes returning to sport-specific activity earlier than 9 months increase their risk of a second ACL injury by up to 7 times more than those who delay return [33]. In this sense, the International Olympic Committee for youth athletes, through an international panel of experts with an expertise in treating and researching pediatric ACL injuries, established that the risk of a second ACL injury following ACL reconstruction is higher during the first 12 post-operative months. Thus, their recommendations include not returning to pivoting sports until at least 12 months following ACL surgery [34]. More conservative proposals based on biological healing and favorable clinico-functional outcomes increase this recommendation up to 2 years. Reasons for that are based on the fact that almost one-third of the younger cohort resuming sports participation will suffer a new episode of ACL injury within the first 24 months after surgery, and that nearly 50% of these re-tears are non-contact injuries, which means being potentially preventable [35]. Research studies have demonstrated that only 11–20% of ACL patients meet all the strength criteria to be cleared for sport between months 9 and 12 after surgery [36,37]. In our cohort, return to sports was individualized depending on the patient’s progression through the rehabilitation program in terms of muscle strength and neuromuscular control, and was encouraged to be achieved after 9 months.

### Strengths and Limitations

This is a novel ACL reconstruction technique with ALL reinforcement which can be a valuable option in skeletally immature patients. It ensures an anatomical position at the tibial ACL footprint, a reliable femoral fixation, additional rotational stability, preserves the vascularization of the hamstring graft and avoids iatrogenic damage to the growth plates. Nevertheless, the findings of our study are subjected to several limitations. First, this is a single-center investigation which represents an audit of retrospectively collected data that might have given rise to biases and inaccuracies. Second, a limited sample size and a short follow-up period were employed, reducing the potential generalizability of this study. Other limitations affecting the external validity of our study include the lack of a control group and randomization, different follow-up periods for different injuries and the gender imbalance in our sample. Though our findings have proven the above procedure to be safe and efficacious, larger, multicenter prospective studies would contribute to the elimination of biases and provide more statistically significant conclusions.

## 5. Conclusions

Our novel ACL reconstruction technique resulted in good clinical outcomes and return to sports in skeletally immature patients, without serious postoperative complications. Therefore, we consider it a useful method for the treatment of these injuries as it achieves an anatomical position at the tibial footprint, reliable femoral fixation, rotational stability, and preserves the vascularization of the graft. However, our investigation is a small-sample, single-center retrospective study with a relatively short follow-up. Long-term, multicenter, randomized controlled studies with wider sample studies could further strengthen our preliminary data.

## Figures and Tables

**Table 1:** General data of patients.

Patient	Gender	Age	Injuries	Side	Treatment	Follow-Up
1	Male	14	ACL + lateral meniscus tear	Right	ACL reconstruction + meniscal suture	50
2	Female	14	ACL	Left	ACL reconstruction	44
3	Male	13	ACL + medial meniscus bucket handle tear	Left	ACL reconstruction + meniscal suture	39
4	Male	14	ACL + medial meniscus tear	Left	ACL reconstruction + meniscal suture	37
5	Male	13	ACL + medial and lateral meniscal tears	Right	ACL reconstruction + meniscal suture	32
6	Male	14	ACL	Right	ACL reconstruction	28
7	Male	13	ACL	Left	ACL reconstruction	25
8	Male	12	ACL	Right	ACL reconstruction	22
9	Male	14	ACL + medial meniscus bucket handle tear	Right	ACL recunstruction + partial meniscectomy	10
10	Male	13	ACL + lateral meniscus radial tear	Left	ACL reconstruction + parcial meniscectomy	9
11	Male	13	ACL	Right	ACL reconstruction	13
12	Male	11	ACL	Right	ACL reconstruction	6

**Table 2:** Clinical and functional outcomes after “over the top” anterior cruciate ligament reconstruction associated with a lateral extra-articular tenodesis. IKDC: International Knee Documentation Committee.

Patient	Lysholm Score	Tegner Test	IKDC Score	KT-1000 Difference	Mobility	Pivot Shift	Lachman	Anterior Drawer	Posterior Drawer
1	100	9	99.4	−1.5	Full ROM	-	-	-	-
2	100	9	96.4	3	Full ROM	-	-	-	-
3	100	9	96.4	1.5	Full ROM	-	-	-	-
4	55	9	59.5	3	Full ROM	slight	-	+1	-
5	100	9	98.2	0	Full ROM	-	+1A	-	-
6	100	9	97.6	3.5	Full ROM	-	-	+1	-
7	100	9	90.5	−1	Full ROM	-	-	-	-
8	86	9	90.5	0	Full ROM	-	-	-	-
9	100	9	99.4	0	Full ROM	-	-	-	-
10	100	9	97.6	2	Full ROM	-	-	-	-
11	100	9	97.6	1	Full ROM	-	-	-	-
12	100	9	96.4	0	Full ROM-	-	-	-	-

## Data Availability

The research data is not publicly available due to privacy or ethical consideration. Once this manuscript is accepted for publication, delinked data without personal privacy could be provided upon reasonable request.
